# Physician and patient perceptions of surgical procedures for osteoarthritis of the knee in the United States, Europe, and Japan: results of a real-world study

**DOI:** 10.1186/s12891-022-05954-x

**Published:** 2022-12-05

**Authors:** N Fukui, PG Conaghan, K Togo, N Ebata, L Abraham, J Jackson, M Berry, JC Cappelleri, H Pandit

**Affiliations:** 1 University of Tokyo, Tokyo, Japan; 2 Leeds Institute of Rheumatic and Musculoskeletal Medicine, University of Leeds & NIHR Leeds Biomedical Research Centre, Leeds, UK; 3 Pfizer Japan Inc, Tokyo, Japan; 4 Pfizer Ltd, Surrey, UK; 5 Adelphi Real World, Bollington, UK; 6 Pfizer Inc, New York, USA; 7 University of Leeds, Leeds, UK

**Keywords:** Knee osteoarthritis, Multinational, Real-world; survey, Surgery

## Abstract

**Background:**

Osteoarthritis (OA) is the most common form of arthritis, with the knee being the joint most frequently affected, and symptomatic knee OA affecting around one quarter of the general population. For patients who do not respond to non-pharmacologic or pharmacologic treatment, surgery is a recommended option. The objectives of this study were to compare the willingness of patients with knee OA to undergo surgery, together with reasons for delaying surgery, and factors affecting successful outcomes.

**Methods:**

A point-in-time survey was conducted in 729 primary care physicians, rheumatologists, orthopedic surgeons, and 2,316 patients with knee OA across three geographical regions: Japan, the United States (US), and Europe (EUR: France, Spain, Italy, Germany, and the United Kingdom), in order to garner their perceptions of knee surgery. Regression models were used to identify factors that might affect patients’ and physicians’ perceptions of surgery, including severity of OA (mild/moderate/severe), number of affected joints, surgery status, and willingness to undergo or delay surgery.

**Results:**

Baseline demographics were similar between US and EUR, although patients in Japan were more likely to be female, older, and only 7% in fulltime employment. We found that few patients with end-stage knee OA, across all regions, but particularly Japan, were willing to undergo surgery (Japan 17%, US 32%, EUR 38%), either through fear, or the lack of awareness of the risk/benefits. Moreover, surgeons are prepared to delay surgery in elderly or unwilling patients, due to their dissatisfaction with the outcome, and may defer surgery in younger patients due to the need for future revision. We also identified a disconnect between physicians, of whom over 80% consider improved functioning to be the most important outcome of surgery, and patients, who seek pain relief (Japan 60%, US 35%, EUR 14%). Since physicians across all regions considered pain reduction to be an indication of surgery success (Japan 27%, US 47%, EUR 43%), this may indicate a need for improved communication to patients on the potential benefits of surgery.

**Conclusion:**

Managing the expectations of patients undergoing surgery remains an important goal in the treatment of knee OA and may help guide physician choice.

## Introduction

OA is strongly age-related and the most common cause of disability in the elderly, limiting their ability to carry out daily tasks and activities, and impacting their overall quality of life [1, 2]. Effective treatment options are limited, and international guidelines for the management of OA recommend initial pharmacologic and non-pharmacologic approaches, followed by appropriate surgical intervention for those who do not achieve adequate pain relief and functional improvement [3–5]. In Japan, non-surgical treatment, such as exercise programs, weight management, and pharmacotherapy, are considered the most appropriate treatment in 90% of patients with knee OA [6].

Surgical management for knee OA involves a spectrum of interventions, ranging from arthroscopic debridement, cartilage regeneration techniques, high tibial osteotomy, to focal resurfacing and unicompartmental or total knee replacement (TKR) [7]. Arthroscopic debridement of the knee may only provide short-term symptom relief [8], while TKR is now one of the most common orthopedic surgeries for patients with end-stage knee arthritis, with reproducible return to daily activities, good survival rates, and overall functional improvement over a longer time period [9].

Although TKR improves quality of life, relieves pain, and improves function, a significant proportion of patients still experience pain, loss of function, deficient muscle strength, and/or reduced walking speed [10]. In addition, if performed at a younger age, the need for revision surgery is significantly higher, which is costly, associated with higher morbidity and mortality and at times sub-optimal clinical outcomes [11].

Worldwide estimates of knee surgery show huge variation across countries, with an average of around 135 TKRs performed per 100,000 population [12]. The variation in the uptake of TKR surgery for patients with OA has been well documented [13]. For example, in Germany, France and Italy, the rate of knee replacement in 2017 was more than twice as high as others, even after age-standardization, with Germany having amongst the highest rates of TKR at 223/100,000 population, and Japan amongst the lowest at 65/100,000 [14], while the prevalence of TKR among the total United States (US) population in 2010 was reported to be 1.52%, higher among women, and increasing with age [15].

Potential reasons for this variation have not been fully explored, although fear of associated morbidity / mortality contributes to the reluctance of some patients with knee OA to undergo surgery [13]. At present, to our knowledge, there are no large multi-national studies that examine physicians’ and patients’ perceptions about surgical management of knee OA.

### Objectives

The objectives of this multi-national survey were to compare the willingness of patients with knee OA to undergo surgery, together with reasons for delaying surgery, and factors affecting successful outcomes across Japan, the US and five major European countries (EUR): France, Spain, Italy, Germany, and the United Kingdom (UK).

## Methods

### Study design

Data for this study were abstracted from the Adelphi Osteoarthritis Disease Specific Program (DSP™), a point-in-time survey conducted from 2017 to 2018 in primary care physicians (PCP), rheumatologists, orthopedic surgeons, and patients with OA across the three geographical regions (Japan, EUR, and the US). Physicians were recruited through publicly available lists, and the data collection setting was in primary care or secondary care rheumatology services (public or private hospitals, clinics, or offices). To be invited to take part in the DSP survey, physicians must have been involved in patient treatment decisions for a minimum of 10 patients with OA per calendar month.

Physicians included in the survey were invited to complete an online patient record form (PRF) questionnaire for 6–10 eligible patients who consulted for routine care. This consisted of information on patient demographics, any previous surgery for OA, type of surgery, success of surgery, how success was defined, and reasons for wishing to delay surgery. Patients were also invited to complete a Patient Self-Completion form (PSC) on a voluntary basis. This included information on their willingness to undergo OA surgery, reasons for not wanting surgery, success of their surgery, and how they defined success. Patients were excluded if they were diagnosed with OA in the hip, ankle and/or foot. All study materials were developed in English before being translated into the local languages by a certified translation agency.

### Data analysis

Since this study was intended to be exploratory, with the aim of generating hypotheses for further research, the analyses were mostly descriptive in nature and no causal relationships could be established with confidence. Categorical variables were described by counts and proportions of respondents, while continuous numerical variables were described by their means and standard deviations [16].

Binary logistic regression models were used to identify statistically relevant differences between binary dependent variables and independent variables, by estimating probabilities using a logistic function [17]. Multinomial logistic regression was used to model nominal outcome variables (three or more categories or levels), in which the log odds of the outcomes were modeled as a linear combination of the predictor variables, with 95% confidence intervals derived via a modified Newton–Raphson algorithm. Relative risk ratios (RRR) were used to describe the probability of an event occurring in one category relative to a control category.

Covariates used in the regression models included region, age, sex, body mass index (BMI), anxiety and/or depression, employment status, current severity, number of affected joints, patient- or physician-reported satisfaction with medication. Stata version 16.1 or later (Stata Statistical Software, College Station, TX: StataCorp LP) was used to perform the analyses; for all regressions, a *p*-value < 0.05 was considered statistically significant.

## Results

A total of 729 physicians (Japan 87, US 153, EUR 489) provided demographic and clinical data for selected patients with OA. The sample included 373 PCPs (Japan 26, EUR 266, US 81), 147 rheumatologists (Japan 11; EUR 101; US 35) and 219 orthopedic surgeons (Japan 60; EUR 122; US 37) who provided data for 2,316 patients (Japan 302, US 527, EUR 1,487). Of these, 1,243 patients completed a PSC form (Japan 230, US 283, EUR 730) which recorded personal perceptions of surgery.

Baseline demographics were similar between the US and EUR with respect to age, gender, habitation, and employment (Table 1). However, patients in Japan were more likely to be female, older, have a lower BMI and different habitation compared to those in the US (Table 1). Only 7% of patients in Japan were in fulltime employment, with almost half being homemakers, and one quarter retired. In comparison, more patients were in fulltime employment in the US and EUR, and approximately half were retired. Patients were diagnosed by physicians with mild (Japan 41%; US 33%; EUR 22%), moderate (Japan 51%; US 47%; EUR 55%), or severe (Japan 8%; US 20%; EUR 23%) OA of the knee (Table 1).

**Table 1:** Patient demographics

	**Japan**	**US**	**EUR**
	***n* = 302**	***n* = 527**	***n* = 1487**
Age, mean (SD)	74.4 (9.4)	64.3 (11.7)	66.6 (11.5)
Female, n (%)	236 (78%)	284 (54%)	866 (58%)
BMI, mean (SD)	24.5 (4.6)	30.2 (7.0)	28.1 (4.8)
**Ethnicity**			
White/Caucasian, n (%)	0 (0%)	388 (74%)	1348 (91%)
Hispanic/Latino, n (%)	0 (0%)	33 (6%)	47 (3%)
Japanese, n (%)	301 (100%)	0 (0%)	0 (0%)
African American, n (%)	0 (0%)	77 (15%)	0 (0%)
**Habitation**			
Lives alone, n (%)	52 (17%)	116 (22%)	335 (23%)
Lives with partner/spouse, n (%)	109 (36%)	354 (67%)	998 (67%)
Lives with family/friends, n (%)	89 (29%)	38 (7%)	91 (6%)
**Employment**			
Working full time, n (%)	22 (7%)	200 (38%)	338 (23%)
Working part time, n (%)	17 (6%)	39 (7%)	78 (5%)
Homemaker, n (%)	149 (49%)	50 (9%)	182 (12%)
Retired, n (%)	68 (23%)	214 (41%)	806 (54%)
Unemployed, n (%)	3 (1%)	20 (4%)	31 (2%)
**Severity of OA**			
Mild, n (%)	124 (41%)	173 (33%)	321 (22%)
Moderate, n (%)	152 (50%)	246 (47%)	815 (55%)
Severe, n (%)	25 (8%)	107 (20%)	341 (23%)

### Surgical history

There were significant differences in surgical history across the regions, with approximately 10% of patients in Japan having undergone previous OA surgery, and a greater proportion in the US and EUR (21% and 17%, respectively, Table 2). There were also some significant differences in procedures across the regions, with TKR being the most frequent intervention (Table 2), but higher usage in patients in Japan compared to the US and Europe (Japan 85%; US 46%; EUR 48%). In contrast, arthroscopic debridement was performed in approximately one third of patients in the US and EUR (US 38%; EUR 28%), but not at all in Japan. Patients with severe OA, as adjudged by their physician, were four times more likely to have had surgery than those at a milder stage (OR 4.09; 95% CI 2.08–8.04, *p* < 0.001, Table 3).

**Table 2:** Surgical history

	**Japan**	**US**	**EUR**	***p*-value**
**History**	***n* = 302**	***n* = 527**	***n* = 1487**	
Previous surgery, n (%)	30 (10%)	113 (21%)	250 (17%)	< 0.001^a^
Willing to have surgery	***n* = 217**	***n* = 274**	***n* = 704**	0.013^a^
Total	36 (17%)	88 (32%)	279 (38%)	0.013 ^a^
Male	9 (25%)	47 (53%)	114 (42%)	
Female	27 (75%)	41 (47%)	156 (58%)	
**Procedure**	***n* = 20**	***n* = 76**	***n* = 164**	
Total knee replacement, n (%)	17 (85%)	35 (46%)	76 (48%)	0.005^a^
Arthroscopic washout/debridement, n (%)	0 (0%)	29 (38%)	45 (28%)	0.004^a^
Synovectomy procedure, n (%)	1 (5%)	2 (3%)	8 (5%)	0.612^b^
Osteotomy, n (%)	0 (0%)	2 (3%)	8 (5%)	0.584^b^
Joint revision, n (%)	1 (5%)	0 (0%)	7 (4%)	0.102^b^
Conversion to joint replacement, n (%)	0 (0%)	2 (3%)	4 (3%)	1.000^b^
Arthrodesis, n (%)	0 (0%)	2 (3%)	3 (2%)	0.776^b^

^a^Chi-squared test

^b^Fisher’s exact test

**Table 3:** Regression data -characteristics (having had surgery vs. not having had surgery)

**Predictor**	**Odds ratio**	**Lower CI**	**Upper CI**	***p*-value**
**Region**				
EUR^a^	1			
US	0.41	0.02	10.01	0.582
Japan	1.22	0.02	98.61	0.929
**Age**	1.00	0.98	1.02	0.764
**Gender**				
Female^a^	1			
Male	1.08	0.76	1.55	0.669
**BMI**	0.99	0.95	1.03	0.637
**Symptoms**				
No anxiety/depression^a^	1			
Anxiety/depression	1.10	0.71	1.70	0.659
**Employment**				
Working (full time & part time)^a^	1			
Retired	1.36	0.78	2.36	0.28
Other	1.48	0.81	2.72	0.20
**Disease severity**				
Mild^a^	1			
Moderate	1.72	0.92	3.21	0.088
Severe	4.09	2.08	8.04	< 0.001
**Physician satisfaction with medication**				
Very satisfied^a^	1			
Somewhat satisfied	0.80	0.45	1.45	0.467
Neither satisfied nor dissatisfied	0.81	0.42	1.55	0.52
Very/Somewhat dissatisfied	0.83	0.39	1.74	0.613

^a^Reference case

### Willingness to undergo surgery, and reasons for delaying surgery

Significantly fewer patients in Japan were willing to undergo surgery than in the US and EUR (Japan 17%, US 32%, EUR 38%, respectively, Table 2; RRR 0.00016; 95% CI 0–1.27 *p* = 0.056, Table 4). Overall, female patients were more willing to agree to surgery than men (RRR 0.68; 95% CI 0.45–1.02 *p* = 0.065, Table 4) while patients with severe OA were almost five times more willing to agree to surgery than patients with milder disease (RRR 4.71;95% CI 2.42–9.17 *p* < 0.001, Table 4).

**Table 4:** Regression data -willingness to have surgery (yes/no)

**Predictor**	**Relative risk ratio**	**Lower CI**	**Upper CI**	***p*-value**
**Region**				
EUR^a^	1			
US	0.04	0.00	1.24	0.066
Japan	0.00	0.00	1.27	0.056
**Age**	0.99	0.96	1.01	0.268
**Gender**				
Female^a^	1			
Male	0.68	0.45	1.02	0.065
**BMI**	1.01	0.97	1.06	0.612
**Symptoms**				
No anxiety/depression^a^	1			
Anxiety/depression	0.69	0.42	1.13	0.143
**Employment**				
Working (full time & part time)^a^	1			
Retired	1.09	0.58	2.05	0.794
Other	0.80	0.38	1.65	0.54
**Disease severity**				
Mild^a^	1			
Moderate	1.64	0.96	2.80	0.07
Severe	4.71	2.42	9.17	< 0.001
**Patient satisfaction with medication**				
Very satisfied^a^	1			
Somewhat satisfied	1.40	0.73	2.71	0.311
Neither satisfied nor dissatisfied	1.75	0.84	3.62	0.134
Very/Somewhat dissatisfied	2.13	0.94	4.82	0.07

^a^Reference case

**Fig. 1:**
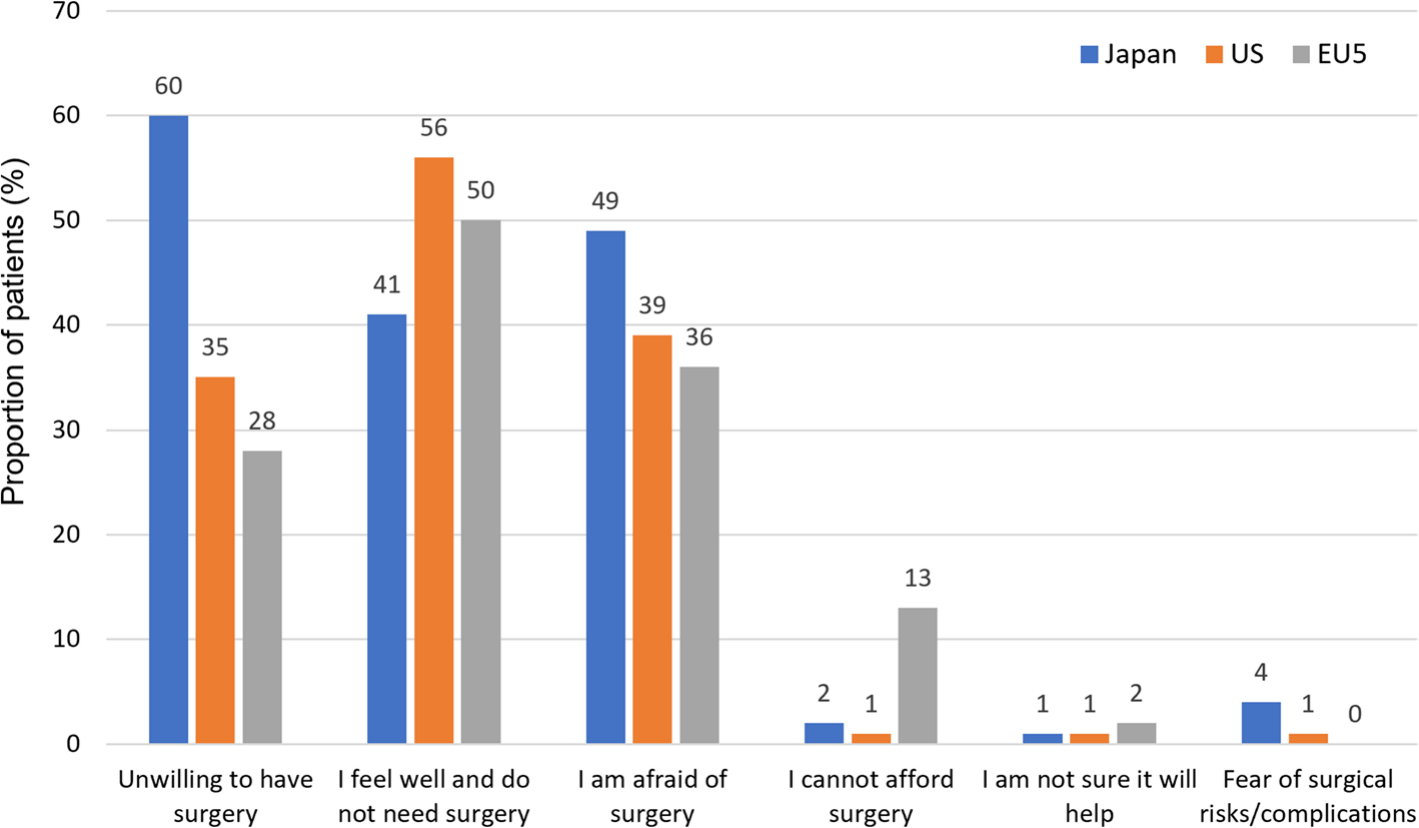
Reasons for patients not agreeing to undergo surgery

**Fig. 2:**
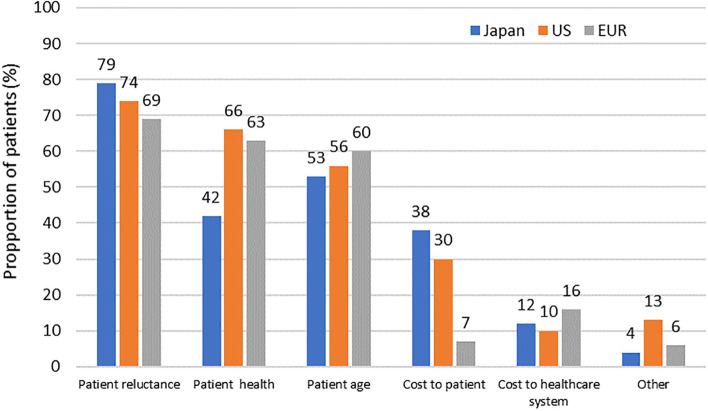
Reasons for physicians delaying surgery

Whilst approximately half of patients reported that they felt well and that they had no need of surgery (Japan 41%, US 56%, EUR 50%; Fig. 1), many patients reported their fear of surgery as a reason for wanting to delay surgery (Japan 49%, US 39%, EUR 36%; Fig. 1) especially women compared with men (OR for male vs. female 0.50 95% CI 0.29–0.89, *p* = 0.017). The cost of surgery was considered to be a potential reason for delaying surgery only by a few patients (13%) in Europe (Fig. 1). This was similarly reflected in physician data, where the majority of physicians across all regions responded that delaying surgery was often the aim when treating their OA patients. Patient reluctance was reported to be the most common reason for delaying surgery across all regions (Japan 79%, US 74%, EUR 69%; Fig. 2) as well as patient health status and age, while the cost to the patient was a consideration by approximately one third of physicians in Japan and the US, but seldom (7%) in EUR physicians (Fig. 2).

### Determinants of recommendation of surgery

The main factors driving a decision to recommend surgical intervention for patients with knee OA were lack of mobility (Japan 18%; US 68%; EUR 53%; Table 5) and degree of pain at rest (Japan 34%; US 29%; EUR 45%; Table 5). Other factors included failure of pharmacotherapy, degree of joint damage, and patient request, which was more frequent in Japan (45%) than in the US or EUR (20% and 16%, respectively; Table 5).

**Table 5:** Main factors in active recommendation of surgery for a patient with OA

	**Japan**	**US**	**EUR**	***p*-value**
	***n* = 94**	***n* = 153**	***n* = 484**	
Lack of mobility	17 (18%)	104 (68%)	258 (53%)	< 0.001^a^
Degree of pain on movement	49 (52%)	70 (46%)	194 (40%)	0.069^a^
Degree of pain at rest	32 (34%)	45 (29%)	220 (45%)	< 0.001^a^
Failure to control condition with drug treatment	30 (32%)	54 (35%)	190 (39%)	0.332^a^
Degree of joint damage	25 (27%)	39 (25%)	151 (31%)	0.327^a^
Patient’s need for independence	26 (28%)	36 (24%)	108 (22%)	0.530^a^
Patient request	42 (45%)	30 (20%)	79 (16%)	< 0.001^a^
X-ray data	25 (27%)	25 (16%)	99 (20%)	0.151^a^
Age of patient	16 (17%)	8 (5%)	49 (10%)	0.011^a^
MRI results	5 (5%)	22 (14%)	42 (9%)	0.038^a^
Patient’s need to continue working	12 (13%)	17 (11%)	38 (8%)	0.206^a^
Patient’s inability to tolerate anti-inflammatories	3 (3%)	2 (1%)	8 (2%)	0.533^b^

^a^Chi-squared test

^b^Fisher’s exact test

### Definition of surgery success

Improved functionality was reported by physicians to be the most common indicator of surgery success across all regions (Japan 81%; US 91%; EUR 86%; Table 6). Physicians in Japan and the US were more likely to consider pain reduction a sign of successful surgery rather than patients being completely pain free (pain reduction vs pain free: Japan 81% vs 34% of physicians; US 76% vs 50%; EUR 59% vs 53%; Table 6). Approximately two thirds of physicians in each region (Japan 68%; US 62%; EUR 66%) considered patient satisfaction to be a successful outcome, while the ability to perform activities of daily living was also considered an important factor by over half of physicians, particularly in the US and EUR (Japan 49%, US 68%, EUR 68%; *p* = 0.002; Table 6).

**Table 6:** Physician and patient considerations of successful outcomes of surgery

	**Japan**	**US**	**EUR**	***p*-value**
**Physician**	***n* = 94**	***n* = 153**	***n* = 484**	
Improved functionality	76 (81%)	139 (91%)	418 (86%)	0.079^a^
Significant pain reduction but not pain free	76 (81%)	117 (76%)	285 (59%)	< 0.001^a^
Patient satisfaction	64 (68%)	95 (62%)	320 (66%)	0.564^a^
Ability to perform all activities of daily living	46 (49%)	104 (68%)	328 (68%)	0.002^a^
Patient is completely pain free	32 (34%)	76 (50%)	258 (53%)	0.003^a^
Reduction in prescribed therapies	19 (20%)	72 (47%)	243 (50%)	< 0.001^a^
Fewer consultations	9 (10%)	18 (12%)	83 (17%)	0.076^a^
**Patient**	***n* = 15**	***n* = 51**	***n* = 63**	
Reduced/less pain	4 (27%)	24 (47%)	27 (43%)	0.373^a^
Improved function/mobility	5 (33%)	12 (24%)	20 (32%)	0.576^b^
No more pain	9 (60%)	18 (35%)	9 (14%)	< 0.001^b^
Feel better/good	0 (0%)	2 (4%)	14 (22%)	0.004^b^

^a^Chi-squared test

^b^Fisher’s exact test

Reduced pain was also the most common determinant of surgery success reported by patients in the US and EUR (Japan 27%; US 47%; EUR 43%; Table 6), although Japanese patients were more likely to define surgery success as having no more pain, than US and EUR patients (Japan 60%; US 35%; EUR 14%, *p* < 0.001; Table 6). Other factors contributing to patients reporting a successful outcome included improved function/mobility and wellbeing (Table 6).

## Discussion

OA of the knee is a relatively prevalent debilitating condition that can progress to a point where the patient’s quality of life is adversely affected due to pain and decreased function. When non-pharmacologic and pharmacologic treatments provide insufficient pain relief and/or improvement in function, surgical options are recommended, the most common being TKR [18]. The attitudes and beliefs of patients with knee OA about surgery are generally based on personal experiences, expectations, and fears, and are influenced by their social environment [19]. A recent systematic literature review found that patients had a fear of surgery, fear of anesthesia, concerns over postoperative pain or complications, and concerns regarding long-term outcomes [13].

In our study, fear was an important reason for postponing surgery, even for patients who received clinical advice to undertake the operation. Another factor that was considered is age, since older patients have been found to be more likely to postpone or refuse surgery, because they feel too old, suffer from severe comorbidities, or prefer other treatment options, such as medication or physical therapy [20]. Conversely, physicians may prefer to delay surgery in younger patients until they are older, in order to avoid the need for revision surgery.

In Japan, OA is a leading cause of years lived with disability, with the average age of patients around 70 years [1], similar to patients with OA in the US [2] and EUR [21]. In our study, the patients in Japan were, on average, older than those in the US and EUR, predominantly female, with milder severity of OA and greater reluctance and fear, possibly driven by concerns around safety risks associated with surgery. The greater reluctance to undergo surgery might also be related to the fact that TKR, which is almost the only surgery performed for knee OA in Japan, is a more invasive procedure that requires a longer hospital stay compared to arthroscopic debridement. Since approximately 20% of patients remain dissatisfied post-TKR, patients who are offered TKR, always have a dilemma about whether to undergo major and invasive surgery or bear the pain. Various non-medical factors and beliefs can contribute to the decision of a patient to proceed with a major surgical intervention. Whilst these concerns are likely to vary widely between patients, we hope that raising awareness of the role that fear plays as a barrier to surgery will encourage physicians to probe this in-depth during discussions with patients, enabling them to address any specific fears in advance.

Physicians across all regions were more likely to report pain reduction as an indication of surgery success than patients, suggesting that, although pain relief and improved physical function should be the main aims of OA surgery, expectations should be explicitly addressed before surgery [22]. Indeed, patients have been reported to opt for premature surgery because of unrealistic expectations of positive outcomes, undervaluation of the risk of negative outcomes, and lack of awareness of alternative treatments [23], which may indicate a need for improved communication to patients on the expected benefits and risks of surgery.

It is interesting to note that no patient in Japan underwent arthroscopic debridement, unlike a significant proportion of patients with knee OA in the US and EUR. Although the overall number of patients undergoing any surgery are small (particularly in Japan), the differences in the trends regarding type of surgical treatment are obvious. Arthroscopic surgery for knee OA was (and still is) often used as a temporary measure to delay joint replacement by performing lavage or debridement to help alleviate OA symptoms. Various studies have confirmed the ineffectiveness of such interventions [24]. Factors other than patient symptoms and severity of knee OA may play a role in the reported variation in practice.

Although no specific single leading factor has been found, patients’ expectations, higher functioning before surgery, lower stage of arthritic disease, complications, poor resolution of pain, and lower improvement in knee function, are more common in dissatisfied patients [25, 26]. Patient satisfaction is thus an important outcome measure because of the well-documented discrepancy between clinician and patient ratings of pain intensity and its impact on quality of life and overall wellbeing [27–29]. Identifying the causes of dissatisfaction is also important in order to improve patient selection for OA surgery, adjust treatment strategies, and to support or treat dissatisfied patients with any residual complaints [22]. There is clearly an unmet need in the management of OA, and future research could focus more on improving patients’ satisfaction with their treatment. Managing the expectations of patients undergoing surgery therefore remains an important goal, recognizing the value of well-informed patients in shared decision making.

### Limitations and strengths

A number of limitations exist given the study methodology. This was a non-interventional study, with physicians providing data on differing numbers of patients depending on the number of patients with knee OA at each site. Moreover, the DSP™ is not a true random sample of physicians or patients, and participation is influenced by willingness to complete the survey, with participants encouraged, but not required, to complete all forms, such that the base sizes fluctuate across different variables [30]. Moreover, patient opinion was based around information from those patients who volunteered opinions and may, therefore not be a true representation of all patients’ feelings about surgery. Finally, the survey addressed OA surgery in general, while many respondents in Japan may have focused specifically on the more common TKR, with debridement being the predominant focus in the US and EUR. Caution should be exercised when interpreting data on surgery in Japan since physicians only provided data on the relatively low number of patients who had undergone surgery.

The strength of the study is that it reflects real-world clinical practice and provides an insight into the acceptance of knee surgery by both physicians and their patients. Since this study involved a relatively high number of physicians from different geographical regions, the sample is likely to be representative of the overall population of patients with facing knee surgery in those countries.

## Conclusions

Although surgery is frequently recommended for patients with knee OA, some patients are reluctant to undergo invasive surgical procedures due to fear of the operation and its outcomes. This was particularly significant in Japan, possibly due to the higher average patient age and the trend for patients to choose non-surgical therapies. Physicians aiming to delay surgery were influenced by patient reluctance, particularly in Japan, while potentially higher costs were only a factor in Europe. Since physicians across all regions were more likely than patients to report pain reduction as an indication of surgery success than patients, this may indicate a need for improved communication to patients on the potential benefits of surgery.

## Data Availability

All data, i.e. methodology, materials, data and data analysis, that support the findings of this survey are the intellectual property of Adelphi Real World. All requests for access should be addressed directly to James Jackson at James.Jackson@adelphigroup.com. James Jackson is an employee of Adelphi Real World.

## Abbreviations

BMI: Body mass index
CI: Confidence interval
DSP: Disease Specific Programme
EUR: Europe
OA: Osteoarthritis
PRF: Patient record form
PSC: Patient self-completion form
RRR: Relative risk ratio
TKR: Total knee replacement
UK: United Kingdom
US: United States
